# New use of an old drug: inhibition of breast cancer stem cells by benztropine mesylate

**DOI:** 10.18632/oncotarget.13537

**Published:** 2016-11-24

**Authors:** Jihong Cui, Maija Hollmén, Lina Li, Yong Chen, Steven T. Proulx, Daniel Reker, Gisbert Schneider, Michael Detmar

**Affiliations:** ^1^ Institute of Pharmaceutical Sciences, Swiss Federal Institute of Technology, ETH Zürich, Zürich, Switzerland

**Keywords:** Prestwick library, NCI DTP-diversity set II, cell-based phenotypic screening, benztropine mesylate, breast cancer stem cells

## Abstract

Cancer stem cells (CSCs) play major roles in cancer initiation, metastasis, recurrence and therapeutic resistance. Targeting CSCs represents a promising strategy for cancer treatment. The purpose of this study was to identify selective inhibitors of breast CSCs (BCSCs). We carried out a cell-based phenotypic screening with cell viability as a primary endpoint, using a collection of 2,546 FDA-approved drugs and drug-like molecules in spheres formed by malignant human breast gland-derived cells (HMLER-shEcad cells, representing BCSCs) and control immortalized non-tumorigenic human mammary cells (HMLE cells, representing normal stem cells). 19 compounds were identified from screening. The chemically related molecules benztropine mesylate and deptropine citrate were selected for further validation and both potently inhibited sphere formation and self-renewal of BCSCs *in vitro*. Benztropine mesylate treatment decreased cell subpopulations with high ALDH activity and with a CD44^+^/CD24^−^ phenotype. *In vivo*, benztropine mesylate inhibited tumor-initiating potential in a 4T1 mouse model. Functional studies indicated that benztropine mesylate inhibits functions of CSCs via the acetylcholine receptors, dopamine transporters/receptors, and/or histamine receptors. In summary, our findings identify benztropine mesylate as an inhibitor of BCSCs *in vitro* and *in vivo*. This study also provides a screening platform for identification of additional anti-CSC agents.

## INTRODUCTION

Breast cancer is the most common cancer in women worldwide, and is also the second leading cause of cancer death in women. Breast cancer patients are treated with cytotoxic, anti-hormonal and immunotherapeutic agents targeting HER-2 in the adjuvant, neoadjuvant and metastatic settings, depending on the molecular and biological characteristics of the cancer. However, drug resistance is a major problem [1] and increasing evidence indicates that a possible cause for treatment failure is the existence of cancer stem cells (CSCs) [2–6].

The CSC hypothesis proposes that a small subpopulation of slow-growing tumor cells have self-renewal ability and drive tumorigenesis, progression and metastasis [7–11]. The differentiation ability of CSCs contributes to tumor cellular heterogeneity and it can give rise to a hierarchy of proliferative and progressively differentiating cells, which can generate the full repertoire of tumor cells including both tumorigenic cells and non-tumorigenic cells [12]. From a therapeutic perspective, the selective targeting of CSCs could be an efficient approach to control cancer growth.

Several agents have been identified that may selectively target CSCs. Salinomycin exhibited inhibitory effects on epithelial-mesenchymal transition (EMT)-induced breast cancer SCs and reduced the CD44^+^/CD24^−^subpopulation [13]. Metformin, a first-line drug used for treating type II diabetes, was reported to selectively kill a chemotherapy-resistant subpopulation of CSCs in an *in vivo* breast cancer model [14]. Dasatinib may preferentially inhibit the growth of breast cancers with an EMT-stem cell-like phenotype, particularly of triple-negative cancers of the basal-like subtype [15].

Due to the fact that the CSC subpopulations in tumors are very small, the collection of large numbers of CSCs that can be used for drug screening is a great challenge. Different strategies have been applied to enrich CSCs, including cell sorting based on cell-surface markers [10], isolation of dye-exclusion side population cells [16, 17], sphere formation [18], resistance to chemotherapeutic compounds [3], EMT induction [19] and high activity of the intracellular enzyme aldehyde dehydrogenase (ALDH) [20, 21]. A combination of different methods for CSC enrichment may enrich for cancer cells at a higher level of cancer hierarchy and be more suitable for drug development [22].

The aims of the present study were to establish a simple, reliable and cost-efficient method to screen for selective CSC-targeting drugs and to identify drug candidates for further preclinical studies and potential clinical development. In an effort to derive sufficient CSCs for primary screening, we used EMT-induced CSCs (HMLER-shEcad cells) [13, 19] and applied the sphere culture technique to enrich CSCs further. We also used immortalized non-tumorigenic human mammary (HMLE cells) adherent cells and spheres as controls [19]. We screened a drug library containing FDA-approved compounds (Prestwick library) and a small chemical library with high structural and chemical diversity (NCI-DTP diversity set II) to identify inhibitors of breast CSCs (BCSCs). We identified nineteen compounds that predominantly inhibited the growth of BCSC-enriched spheres, without major influence on normal stem cell -enriched spheres. One group of compounds with the same chemical core structure (benztropine mesylate and deptropine citrate) was identified and further analyzed with regard to the inhibition of functional properties of CSCs *in vitro* and *in vivo*.

## RESULTS

### Mammospheres generated from HMLER-shEcad BCSCs

A major challenge in cell-based phenotypic screening is the limited number of CSCs in cancer cell cultures. To increase CSC numbers, we generated mammospheres from EMT-induced CSCs (HMLER-shEcad cells) and examined whether this might further enrich CSCs compared to adherent culture conditions. CSCs mostly maintain quiescence or are slow-cycling [23]. The HMLER-shEcad spheres demonstrated a significant decrease in proliferation compared to adherent HMLER-shEcad cells (Supplementary Figure S1A; two-way ANOVA, *p*<0.001). HMLER-shEcad spheres also exhibited more resistance to both paclitaxel and doxorubicin than adherent HMLER-shEcad cells (Supplementary Figure S1B). ALDH is used as a biomarker to identify and characterize the BCSC phenotype [24]. FACS data indicated a higher percentage of ALDH^+^ cells in HMLER-shEcad spheres than that in adherent cells (sphere *vs* adherent cells: 6.4±1.01% *vs* 1.5±0.155%, *p*<0.01) (Supplementary Figure S1C-D). Gene expression measurements by qRT-PCR showed that expression of BCSC related genes, including *CD44*, *ALDH1*, *CD133*, *SLUG*, *FOXC2* and *OCT4* was increased in HMLER-shEcad spheres compared with the adherent cells (Supplementary Figure S1E and Supplementary Table S1).

### Identification of compounds with specific inhibition of spheroid CSCs via cell-based phenotypic screening

**Figure 1:**
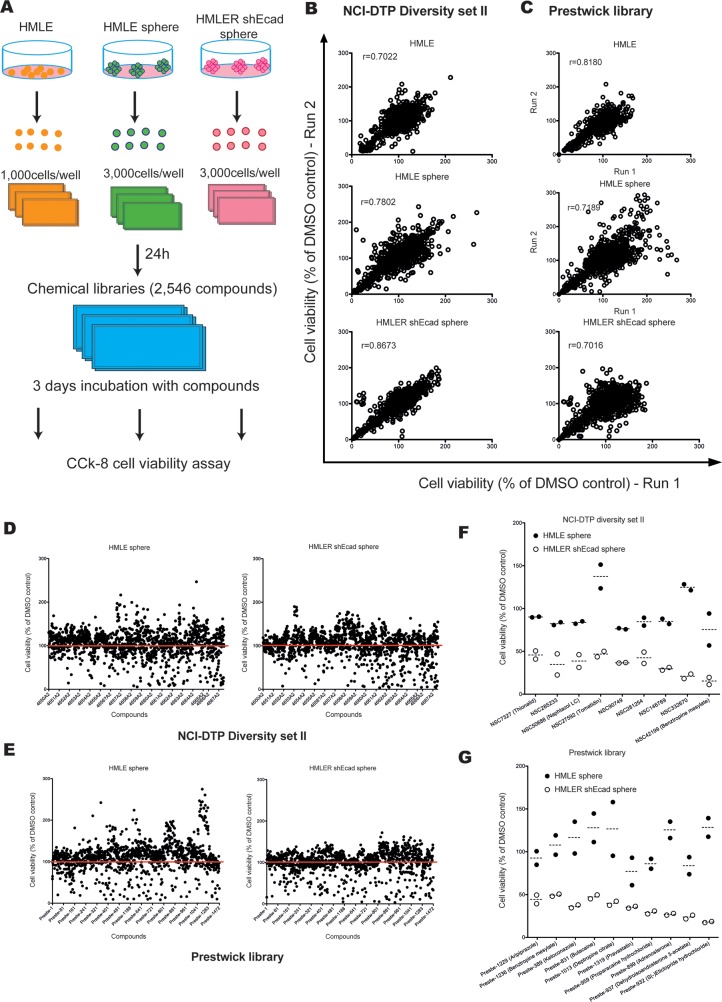
Chemical screening for compounds that selectively inhibit the viability of HMLER-shEcad spheres **A.** Schematic overview of the chemical library screening strategy. **B-C.** Replicate correlation plots of raw values from two replicates of the same compounds in HMLE and HMLER-shEcad adherent cells and spheres, respectively, showing good agreement and suggesting overall good reproducibility. **D-E.** Summary of the cell viability of HMLE and HMLER-shEcad spheres, with all compounds from NCI-DTP diversity set II and Prestwick library. **F-G.** Nineteen candidate compounds were identified by chemical library screening based on cell viability assays.

The above results confirmed that a subpopulation of cells with CSC properties became enriched during mammosphere formation. Therefore, we hypothesized that compounds with a selective inhibition of the HMLER-shEcad spheres might have inhibitory activity on CSCs. For the compound library screening, we first cultured HMLE cells and HMLER-shEcad cells in suspension with SCM to generate sufficient spheres for screening. The primary spheres were dissociated and used to generate subsequent sphere generations, which were used in the screening platform (from the third to the fifth generation). Cells from each cell line were seeded in 96-well plates, allowed to proliferate for 24 h, treated with the compounds of the chemical libraries at 10 μM, and assayed for cell viability after 3 days of incubation (Figure 1A). The screening of 2,546 small molecules was done in two independent experiments with a very high inter-assay correlation (Figure 1B–1C, *r*>0.7). Thus, the protocol enabled consistent generation of high quality sample spots, which was necessary to ensure that sufficient precision in determining deficient samples was achieved and the risk of producing false negative hits was minimized.

**Figure 2:**
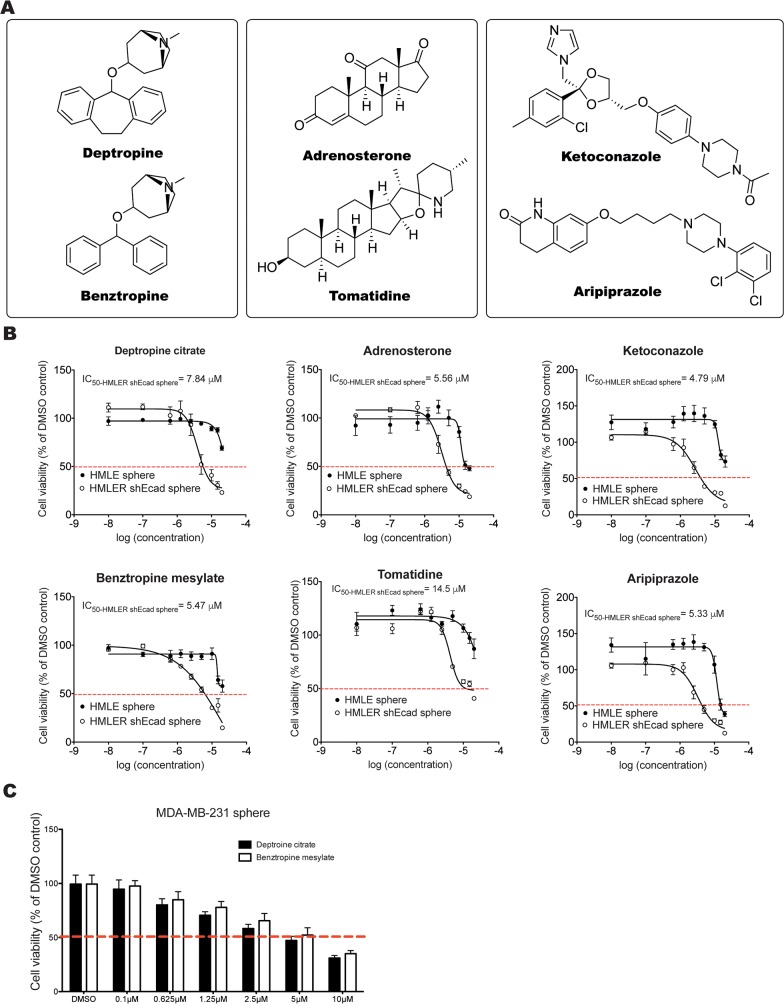
Identification and validation of compounds that exhibit selective inhibitory effects on HMLER-shEcad spheres **A.** Three groups of active compounds with related chemical core structures were identified: Group 1: deptropine (Prestw-1013: deptropine citrate) and benztropine (NSC42199/Prestw-1236: benztropine mesylate); Group 2: adrenosterone (Prestw-899) and tomatidine (NSC27592); Group 3: aripiprazole (Prestw-1229) and ketoconazole (Prestw-389) **B.** Dose-response curves of HMLE spheres and HMLER-shEcad spheres treated with selected compounds. **C.** Cell viability of MDA-MB-231 spheres treated with different concentrations of deptropine citrate and benztropine mesylate for 72 h. Data are expressed as mean±SD.

Approximately 6.0% (152 of 2,546) of the test compounds reduced the viability of HMLER-shEcad spheres by more than 50% (Figure 1D–1E). Out of these, nine compounds from the NCI-DTP diversity Set II (hit ratio: 0.66%) (Figure 1F) and ten compounds from the Prestwick library decreased the viability of control HMLE spheres by 30% or less (hit ratio: 0.80%) (Figure 1G). Among these nineteen hits, three groups of compounds with the same chemical core structures were identified. NSC42199 (from NCI-DTP diversity Set II) and Prestw-1236 (from Prestwick library) are benztropine mesylate. Prestw-1013 (deptropine citrate) and benztropine mesylate share the same chemical core structure (diphenylmethane), as do Prestw-389 (ketoconazole) and Prestw-1229 (aripiprazole) (phenylpiperazine), as well as Prestw-899 (adrenosterone) and NSC27592 (tomatidine) (dimethyldecahydronaphthalene) (Figure 2A). Cell viability was further assessed over a wide range of doses to calculate the half-maximal inhibitory concentration (IC_50_) for each compound. The dose-response curves demonstrated that the cell viability of HMLER-shEcad spheres was inhibited more potently than that of HMLE spheres using the selected compounds (Figure 2B).

Considering the fact that NSC42199 (benztropine mesylate, from NCI-DTP diversity Set II), Prestw-1013 (deptropine citrate) and Prestw-1236 (benztropine mesylate from Prestwick library) were identified from two different libraries, as well as their preferential inhibitory effects on HMLER-shEcad spheres, we focused our investigations on these compounds. We next investigated their effects on the cell viability of spheres induced from two other CSC-enriched breast cancer cell lines, namely human MDA-MB-231 cells and murine 4T1-luc2 cells [25, 26]. The IC_50_ values of benztropine mesylate and deptropine citrate for MDA-MB-231 spheres were ~5 μM (Figure 2C). For 4T1-luc2 spheres, the IC_50_ value of benztropine mesylate was around 5 μM (Supplementary Figure S2).

### Deptropine citrate and benztropine mesylate suppress mammosphere formation and self-renewal capacities of BCSCs in vitro

**Figure 3:**
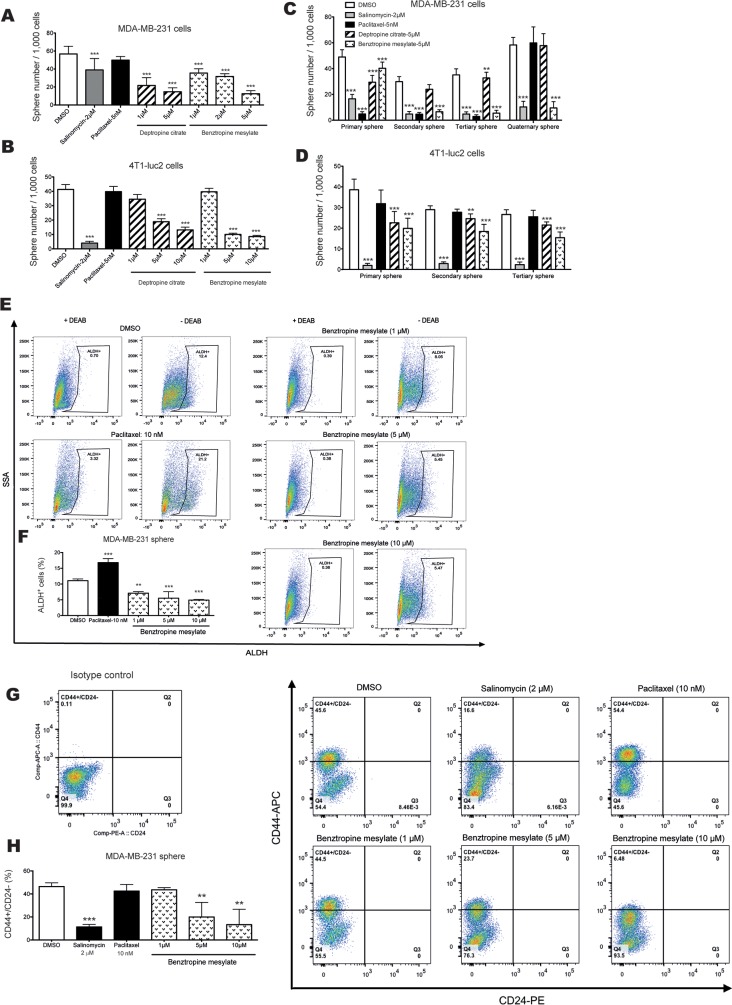
Inhibitory effects of benztropine mesylate on BCSC properties *in vitro* **A-B.** Mammosphere formation assays: Number of mammospheres (diameter > 50 μm) from 1,000 MDA-MB-231 or 4T1-luc2 cells which were treated with different concentrations of deptropine citrate, benztropine mesylate, salinomycin or paclitaxel for 6 days was counted. **C-D.** Self-renewal assays: Adherent cells were pretreated with or without the compounds at indicated concentrations for 4 days and mammosphere formation was evaluated in sequential sphere generations without any treatment. Data are expressed as mean±SD (n=6). FACS analysis of the expression of CSC markers (ALDH^+^ and CD44^+^/CD24^−^) in MDA-MB-231 spheres with or without benztropine mesylate treatment. MDA-MB-231 spheres were treated with benztropine mesylate (1, 5 or 10 μM), salinomycin (2 μM), paclitaxel (10 nM) or DMSO for 6 days. Single cell suspensions were used for FACS analysis. Representative data for ALDH^+^ **E.** and CD44^+^/CD24^−^ **G.** populations in benztropine mesylate-treated MDA-MB-231 spheres show a reduction compared with DMSO-treated cells. DEAB was used to inhibit the reaction of ALDH with the ALDEFLUOR reagent, providing a negative control. The proportions of ALDH^+^ **F.** and CD44^+^/CD24^−^ **H.** cells are shown as mean±SD. Experiments (n=3) were conducted in triplicate. **p<0.01, ***p<0.001 compared with DMSO control (one-way ANOVA).

The ability to form mammospheres is correlated with the frequency of CSCs and progenitor cells in tumor cell lines. Thus, we next analyzed the effects of different concentrations of deptropine citrate and benztropine mesylate on mammosphere formation of MDA-MB-231 and 4T1-luc2 cells. Paclitaxel served as a conventional chemotherapy drug control, whereas salinomycin served as a positive control for selectively targeting CSCs [13]. The mammosphere growth in SCM with or without compounds was observed after 6 days. In MDA-MB-231 cells, deptropine citrate and benztropine mesylate reduced the size as well as the number of mammospheres significantly in a dose-dependent manner (Figure 3A and Supplementary Figure S3A, *p*<0.001). In 4T1-luc2 cells, treatment with 5 or 10 μM, but not 1 μM, of the compounds had a significant inhibitory effect on the number and size of the mammospheres (Figure 3B and Supplementary Figure S3B, *p*<0.001). In contrast, paclitaxel had no major effect on the number of spheres in both cell lines (Figure 3A–3B). The same effect was also found in HMLER-shEcad (data not shown). The inhibitory effects of different concentrations of the compounds on sphere formation corresponded to their effects on cell viability (Supplementary Figure S3C-D).

The ability of self-renewal is a unique characteristic of stem cells. We tested the ability of MDA-MB-231 cells and 4T1-luc2 cells to form subsequent sphere generations in suspension (without treatment) after a 4-day pretreatment of the cells with the selected compounds under adherent conditions. The sphere forming efficiency of MDA-MB-231 cells in different generations was markedly suppressed by pretreatment with 5 μM deptropine citrate and benztropine mesylate, as compared to DMSO (Figure 3C). Moreover, a significant inhibitory effect on sphere formation by 5 μM benztropine mesylate was maintained even in the quaternary spheres, suggesting that treatment with benztropine mesylate reduced the stem cell-like subpopulation, and thus prevented the recovery of sphere formation (Figure 3C). A similar effect was seen for the sphere formation of 4T1-luc2 cells after pretreatment with the compounds (Figure 3D). Thus, the compounds had an apparent effect on the self-renewal capability of breast cancer cells, which persisted after drug withdrawal. Salinomycin potently inhibited sphere formation of both cell lines in all generations, whereas paclitaxel showed inhibition in MDA-MB-231 cells but not in 4T1-luc2 cells.

### Benztropine mesylate decreases the percentage of breast cancer cells expressing CSC markers

In light of the above data, we focused further on the anti-CSC properties of benztropine mesylate. To confirm that benztropine mesylate targets the CSC subpopulation, we analyzed the expression of the prospective BCSC marker combination CD44^+^/CD24^−^ and of ALDH after benztropine mesylate treatment. Incubation with different concentrations of benztropine mesylate resulted in a dose-dependent reduction of the cell percentage with high ALDH activity in MDA-MB-231 spheres, SKBR3 cells and 4T1-luc2 cells (Figure 3E–3F and Supplementary Figure S4). The proportion of ALDH^+^ cells was 11.1% in the DMSO-treated group, and it decreased to 7.1, 5.5 and 4.9% after 6 days treatment with 1, 5 and 10 μM benztropine mesylate, respectively (Figure 3E–3F). In contrast, paclitaxel (10 nM) increased the percentage of ALDH^+^ cells (Figure 3F; 16.8±1.28%, *p*<0.001). The percentage of the CD44^+^/CD24^−^ subpopulation was significantly decreased when MDA-MB-231 spheres were exposed to 5 μM (20.0±12.64%) and 10 μM benztropine mesylate (13.3±13.36%) for 6 days, compared to DMSO-treated cells (46.5±3.30%, *p*<0.01, *n*=3) (Figure 3G–3H). Salinomycin significantly reduced the CD44^+^/CD24^−^subpopulation to 11.3% (±2.21%, *p*<0.001), whereas paclitaxel had no influence on the percentage of the CD44^+^/CD24^−^ subpopulation in MDA-MB-231 spheres (42.5±3.28%, *p*>0.05).

### Benztropine mesylate improves the efficiency of chemotherapy in vitro

**Figure 4:**
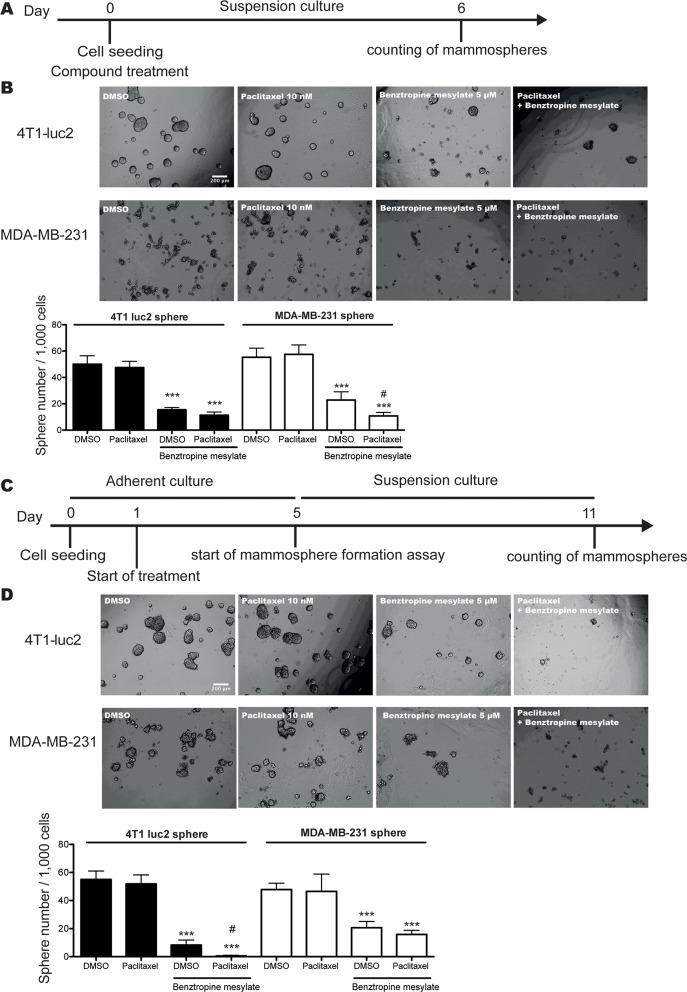
Benztropine mesylate treatment improves the efficiency of chemotherapy *in vitro* **A.** Schematic representation of the experimental approach taken to quantify the mammosphere formation efficiency of 4T1-luc2 and MDA-MB-231 cells by combination treatment with benztropine mesylate (5 μM) and paclitaxel (10 nM) or as single agent treatment. **B.** Representative images and quantification of mammosphere numbers after 6 days. **C.** Schematic representation of the experimental approach taken to quantify the mammosphere formation efficiency of pretreated-4T1-luc2 cells and MDA-MB-231 cells by combination treatment with benztropine mesylate and paclitaxel or by single agent treatment. **D.** Representative images and quantification of mammosphere numbers after 6 days. Data are expressed as mean±SD (*n*=6). ***p<0.001 compared with DMSO control (one-way ANOVA); #*p*<0.05 compared with benztropine mesylate group.

Accumulating evidence indicates that CSCs are largely chemotherapy-resistant [3]. It has been proposed that combined chemotherapy and anti-CSC treatment may improve the efficacy of standard chemotherapy [2]. To investigate this hypothesis, 1,000 4T1-luc2 cells or MDA-MB-231 cells were grown in suspension with SCM in the presence of 10 nM paclitaxel alone, 5 μM benztropine mesylate alone or both drugs combined. After 6 days, compared to DMSO (sphere number/well: 4T1-luc2 spheres: 50.0±6.45; MDA-MB-231 spheres: 55.3±6.83) or paclitaxel (4T1-luc2 spheres: 47.5±4.76; MDA-MB-231 spheres: 57.5±7.18), the combination treatment inhibited the sphere formation significantly (4T1-luc2 spheres: 11.3±2.34; MDA-MB-231 spheres: 10.7±2.73; *p*<0.001). Compared to benztropine mesylate alone (MDA-MB-231 spheres: 22.8±6.27), the combination treatment decreased sphere formation efficiency by 53.1% in MDA-MB-231 spheres (*p*<0.05, Figure 4A-4B).

We next pretreated adherent 4T1-luc2 cells or MDA-MB-231 cells with 10 nM paclitaxel alone or combined with 5 μM benztropine mesylate for 4 days and performed mammosphere formation assays. Compared to DMSO (sphere number/well: 55.0±6.07) or paclitaxel (51.8±6.40) alone, the combination treatment significantly reduced the sphere formation of 4T1-luc2 cells (0.50±0.548), which was more efficient than benztropine mesylate alone (8.33±3.50, *p*<0.05). For MDA-MB-231 cells, both benztropine mesylate alone (sphere number/well: 20.7±4.41) and the combination treatment (15.8±2.93) impaired the sphere formation by >57%, compared to DMSO (47.8±4.54) and paclitaxel alone (46.5±1.23, *p*<0.001). There was no significant difference between benztropine mesylate and combination treatment (Figure 4C–4D).

### Benztropine mesylate inhibits tumor-initiating potential in vivo

To test whether benztropine mesylate might have any anti-CSC activity *in vivo*, we used the gold-standard assay for CSCs, the limiting dilution assay. 4T1 spheres were maintained in SCM and treated with benztropine mesylate or DMSO *in vitro* for 6 days. Single cell suspensions isolated from pretreated-spheres were prepared and injected in serial limiting dilutions (10 - 1,000 cells) into Balb/c mice, which were monitored for subsequent tumor formation for four weeks. We observed that benztropine mesylate pretreatment resulted in a significant reduction in the tumor-initiating potential relative to the DMSO group (Table 1). We further performed an ELDA (extreme limiting dilution assay) to evaluate the effect of benztropine mesylate on the CSC frequency. The repopulating frequency of CSCs was 1 of 218 for benztropine mesylate treatment and 1 of 9 for DMSO control in 4T1 cells. The difference in CSC frequency between the two groups was significant (*p*<0.001, Table 1). We next treated Balb/c mice bearing 4T1 breast tumors with benztropine mesylate (1.5 mg/kg) or 0.9% saline for 3 weeks. Both tumor size and tumor weight were significantly reduced after benztropine mesylate treatment, as compared to the saline treated control group (Supplementary Figure S5A-B). There was no difference in body weight between the treatment groups (Supplementary Figure S5C).

**Table 1:** Tumor incidence in limiting dilution assay

Tumor incidence/injection				
Cells Injected	1,000	100	10	Estimated cancer stem cell frequency with confidence intervals (95%)
4T1-DMSO	10/10	10/10	7/10	1 in 9 (4-18)
4T1-Benztropine mesylate	8/10	7/10	5/10	1 in 218 (100-479)***

4T1-luc2 spheres pretreated with benztropine mesylate or DMSO were dissociated into single-cell suspensions and injected into the mammary fat pads of mice in limiting dilution (10; 100; 1,000). Tumor formation was observed for four weeks following inoculation. BCSC frequency was calculated using ELDA.

### Benztropine mesylate partially inhibits the CSC properties through acetylcholine receptors, dopamine transporters/receptors and/or histamine receptors

**Figure 5:**
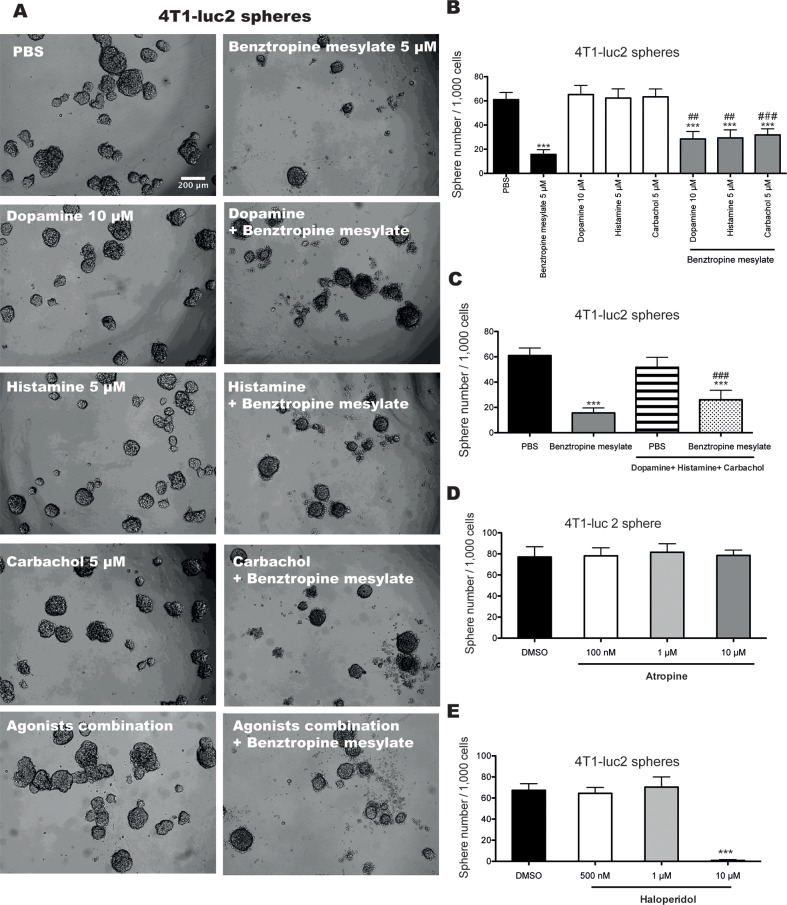
Benztropine mesylate partially impairs mammosphere formation of breast CSCs through acetylcholine receptors, dopamine receptors/transporters and histamine receptors Representative images **A.** and quantification of mammosphere formation efficiency **B.** of 1,000 4T1-luc2 cells co-treated with benztropine mesylate (5 μM) and dopamine (10 μM), histamine (5 μM) or carbachol (5 μM) alone or with an agonist combination (dopamine + histamine + carbachol) for 6 days. Quantification of mammosphere numbers from 1,000 4T1-luc2 cells treated with either the muscarinic receptor antagonist atropine **D.** or the dopamine receptor antagonist haloperidol **E.** for 6 days. Data are expressed as mean±SD (*n*=6). **p<0.01, ***p<0.001 compared with DMSO control (One-way ANOVA).

Benztropine mesylate is used clinically for the management of Parkinson's disease and its pharmacological effects are thought to result from its anticholinergic activity [27]. However, benztropine mesylate is also a centrally acting anti-histamine [28] and dopamine re-uptake inhibitor [29]. To determine which, if any, of these activities play a role in the inhibition of CSC properties, we evaluated the ability of selective agonists of muscarinic acetycholine receptors (mAChRs), nicotinic acetylcholine receptors (nAChRs) (carbachol), dopamine receptors (dopamine) or histaminergic receptors (histamine) to reduce the inhibitory activity of benztropine mesylate on mammosphere formation of BCSCs. The benztropine mesylate-induced inhibition of mammosphere formation in 4T1-luc2 cells was significantly reduced in the presence of carbachol, histamine and dopamine, as well as after combination treatment (Figure 5A–5C, *p*<0.01). Carbachol and histamine also significantly reduced the inhibitory effect of benztropine mesylate on MDA-MB-231 sphere formation (Supplementary Figure S6A-S6B, *p*<0.05). The combination treatment with carbachol, histamine and dopamine partially but not completely blocked the inhibitory effects of benztropine mesylate on mammosphere formation of BCSCs.

We then evaluated a panel of antagonists for these receptors, including the dopamine receptor antagonist haloperidol, the histaminergic receptor antagonist pyrilamine, and the acetylcholine receptor antagonists atropine, hexamethonium bromide and pancuronium on mammosphere formation of 4T1-luc2 cells (Figure 5D–5E and Supplementary Figure S6C-S6E) and MDA-MB-231 cells (Supplementary Figure S6F-S6J). We found that 10 μM of haloperidol inhibited mammosphere formation of 4T1-luc2 cells. The mammosphere formation of MDA-MB-231 cells was also significantly reduced when cells were incubated with 10 nM of pyrilamine or different doses of haloperidol (500 nM, 1 and 10 μM).

To further probe the potential mechanisms of action, we applied the SPiDER protocol [30]. The software successfully ranked known on- and off-targets of the compound among the high confidence predictions (*p*<0.05, Table S2). These results suggest that neurotransmitter receptors may play important roles for the inhibitory effects of benztropine on BCSCs (nicotinic acetylcholine receptor agonist: *p*=0.005; muscarinic acetylcholine receptor antagonist: *p*=0.023; histamine receptor: *p*=0.016; dopamine receptor antagonist: *p*=0.03).

In a next step, we determined the mRNA expression levels of distinct acetylcholine receptors in sphere-forming and adherent HMLER shEcad, HMLER shCtrl and HMLE cells. We found that the mRNA expression levels of α2-nAChR (*CHRNA2*), α5-nAChR (*CHRNA5*), α6-nAChR (*CHRNA6*), α9-nAChR (*CHRNA9*), α10-nAChR (*CHRNA10*) and β1-nAChR (*CHRNB1*) were higher in HMLER shEcad than in HMLER shCtrl cells (*CHRNA5*: >4-fold; *CHRNA9*: >12-fold; *CHRNB1*: >4-fold; *CHRNA2*, *6* and *10*: ~2-fold) (Supplementary Figure S7A). The expression of *CHRNA9* was 17.4-fold higher in sphere-forming HMLER shEcad cells than in adherent HMLER shEcad cells (Supplementary Figure S7B). Importantly, *CHRNA9* mRNA was more strongly expressed (126.8-fold) in HMLER shEcad spheres than in immortalized, non-tumorigenic HMLE spheres (Supplementary Figure S7C).

## DISCUSSION

The existence of CSCs has been reported across a range of hematological as well as solid malignancies, and these cells display the capacity for self-renewal and differentiation, which are critical for tumor initiation, progression, metastasis and recurrence [31]. The CSC model not only provides an explanation for the failure of conventional cancer therapies that target proliferating tumor cells, but also provides an important drug target in cancer [3, 4, 8]. Identification of agents that selectively inhibit the traits of BCSCs has therefore become a key goal in the challenge to improve the efficacy of cancer therapy.

In the present study, we screened two small molecule libraries by using a 96-well plate spheroid-derived CSC growth assay. We used HMLER-shEcad spheres as a model for CSCs. It has been previously shown that HMLER-shEcad cells are enriched with EMT-induced CSCs. These cells are transformed to have a mesenchymal phenotype (a hallmark of CSCs) by down-regulation of E-cadherin, producing a high percentage of a CD44^+^/CD24^−^ population [19]. Dontu et al. indicated that suspension mammospheres are enriched in early progenitor/stem cells and are able to differentiate and generate complex functional structures in a reconstituted 3D-culture system with SCM [18]. Our study combined the two CSC enrichment methods and generated HMLER-shEcad spheres. We found that HMLER-shEcad spheres showed a higher population of slow-cycling cells, a higher percentage of cells with high ALDH activity and increased chemotherapy-resistance, compared with HMLER-shEcad adherent cells. These results indicated that HMLER-shEcad spheres contained a higher proportion of BCSCs. Additionally, HMLER-shEcad cells cultured under adherent conditions in differentiation medium showed a cellular hierarchy with tumorigenic and non-tumorigenic cells, which is an important character of CSCs. Based on CD44 and CD24 expression profiles, distinct cell populations of HMLER-shEcad cells were identified by FACS (data not shown), and previous studies found that the tumorigenic properties of distinct populations with distinct CD44 and CD24 phenotypes were different [10].

Several investigators have aimed to identify compounds that specifically inhibit the CSC-related molecular properties or kill CSCs directly. For example, Marx et al. screened the NCI-DTP diversity set II library and identified four compounds capable of selective silencing ErB2 transcription in breast cancer cells [32]. Three inhibitors (loxapine, pimozide and acacetin) of the ABC transporters (ABCB1, ABCC1 and ABCG2 transporters), which are highly expressed in chemoresistant CSCs, were identified by high-throughput FACS screening of the Prestwick library [33]. Sun et al. performed a cell-based screening using the Prestwick library to identify potential inhibitors of survivin in prostate cancer cells; survivin is a broadly expressed tumor antigen associated with CSCs [34]. Because CSC populations are very complex and multiple CSC pools exist within individual tumors [35], we designed the screening platform based on the function of CSCs, instead of just based on a single CSC-related molecular property.

A major challenge for high-throughput screening is to isolate and scale up sufficient amounts of CSCs. Other CSC enrichment methods based on cell surface markers, Hoechst dye exclusion, or cell auto-fluorescence, are often time- and money-consuming. It is also unclear whether any of these approaches can be used reliably and routinely to enrich CSCs across all subtypes. The spheroid technique used here allows the production of large amounts of CSCs and therefore enables the availability of these cells for screening. Additionally, this assay allows the exclusion of compounds that inhibit not only CSCs, but also NSCs, since NSCs share many properties with CSCs. In fact, salinomycin was found to inhibit BCSC properties but it also exhibited equal toxicity to NSCs *in vitro*, with potential implications for its safety profile [36]. To validate the selectivity of candidate compounds for CSCs, we used HMLE adherent cells and spheres as controls, which contain distinctive and discrete naturally present subpopulations of stem-like and non-stem-like cells [19, 37]. Both HMLE adherent cells and spheres control conditions were used to eliminate compounds with general toxicity for normal breast cells and stem cell-like cells as well as to eliminate the compounds with inhibitory effects due to the suspension culture system. In summary, this model provides a convenient strategy for compound screening, but there are also some limitations: As the model is a specialized screening system and does not represent a specific type of breast cancer, further functional assays using defined human or mouse breast cancer cell lines are needed for confirmation of the screening results.

We used a small-molecule collection consisting of 2,546 compounds from two chemical libraries including the NCI-DTP diversity set II and the Prestwick library for screening. The NCI-DTP diversity set II is an uncharacterized compound library, which provides the possibility of identifying novel lead candidates, with the drawback that the active mechanisms of these compounds are largely unknown. In contrast, the compounds from the Prestwick library are already FDA-approved, well-characterized drugs, which may render the translation of discoveries from the basic laboratory to the clinical application more easily. Also, the already known mechanisms of action of the potential hit compounds may provide some hints for the exploration of the mechanism of their new function.

Based on this robust cell-based screening method, we identified nineteen compounds, including three groups of compounds with related chemical core structures, preferentially targeting the viability of HMLER-shEcad spheres but not HMLE adherent cells and spheres. We focused on characterizing the anti-CSC properties of the compounds deptropine citrate and benztropine mesylate, which have the same chemical core structure. Deptropine citrate, a well-known H1-histamine receptor antagonist and muscarinic receptor antagonist, showed inhibitory effects on cell viability and mammosphere formation of BCSCs, but it did not inhibit the self-renewal capacities of MDA-MB-231 cells. Benztropine mesylate significantly inhibited mammosphere formation and self-renewal of BCSCs. It also decreased the ALDH^+^ and CD44^+^/CD24^−^ CSC subpopulations. Previous studies showed that these markers both enrich for stem cells, however the populations do not appear to correlate highly with each other [38, 39]. Our results indicated that benztropine mesylate could inhibit CSCs with distinct phenotype. Additionally, our *in vivo* studies revealed that benztropine mesylate inhibited the tumor-initiating potential significantly and decreased the CSC frequency. Thus, benztropine mesylate is a potential anti-CSC drug candidate that can alter tumorigenic properties. However, considering the complexity and heterogeneity of human cancers, many preclinical animal models fail to predict the clinical efficacy of novel anti-cancer agents. Further functional research studies using humanized mouse models, such as patient-derived xenograft models [40] or mice with humanized immune system or mammary microenvironment [41] would be helpful to support a potential clinical translation.

Benztropine mesylate is a centrally acting anticholinergic agent for the treatment of Parkinson's disease [27]. In a multiple sclerosis mouse model, benztropine mesylate induced the differentiation of oligodendrocytes through M1 and M3 muscarinic receptors and enhanced re-myelination [42]. Benztropine mesylate also acts as an anti-histamine [28] and a dopamine re-uptake inhibitor [29], and as an allosteric antagonist of the human D2 dopamine receptor (Pubchem BioAssay: AID 485344). In the present study, the pharmacological data indicated that benztropine mesylate partially inhibited the BCSC properties through acetylcholine receptors, dopamine receptors/transporters and/or histamine receptors, even though more detailed follow-up studies might be needed to investigate the relative contribution of these pathways. A previous study reported that thioridazine, an antagonist of the dopamine receptor, impairs human somatic CSCs capable of *in vivo* leukemic disease initiation by inducing differentiation to overcome neoplastic self-renewal, while having no effect on normal blood stem cells [43]. Haloperidol, which exhibits high affinity dopamine D2 receptor antagonism and slows receptor dissociation kinetics [44], inhibited mammosphere formation of BCSCs markedly in our study. Thus, our findings further indicate that dopamine receptors play an important role in mediating BCSC functions, and indicate that dopamine receptors might represent potential CSC markers in breast cancer. Further investigation is required to better understand the connection of dopamine receptor signaling and CSC biology in human cancers. The comparison of acetylcholine receptor expression levels indicates that CSCs have increased acetylcholine receptor expression levels, rendering them more sensitive to inhibition by benztropine mesylate. Future studies are needed to dissect the specific role of α9-nAChR for the biological properties of CSCs.

Other, yet unidentified pathways might also be involved in the anti-CSC effects of benztropine mesylate. A preliminary analysis of the topological pharmacophore feature pattern (CATS2 descriptor) [45] of benztropine mesylate suggested potential activity as a CCR5 antagonist. Increasing evidence indicates that CCL5 and CCR5 are overexpressed in breast cancer, and CCR5 antagonists block metastasis of breast cancer [46, 47]. However, in the DRUGMATRIX screen (https://ntp.niehs.nih.gov/drugmatrix), benztropine mesylate was reported to not posses a high-affinity effect on CCR5 (<50% inhibition at 10 μM ligand concentration). The algorithm analysis by SPIDER software suggested that the compound could be modulating the Akt or Wnt pathways (through Akt or Casein Kinase 1 binding, *p*=0.04, Table S2), which are associated with BCSCs [48–50]. This theoretical analysis points to motivated macromolecular targets of benztropine mesylate in the context of cancer, which deserve further attention.

## CONCLUSIONS

Selective targeting of CSCs offers promise for a new generation of cancer therapeutics. In this study, we developed a cell-based phenotypic screening platform for the identification of CSC-specific inhibitors that have only minor effects on normal stem cells. Benztropine mesylate was identified as a novel potential anti-CSC inhibitor by *in vitro* and *in vivo* assays, thus revealing a novel usage for a known drug that could be readily translated to further preclinical and clinical development. The screening platform established here could also be applied for larger-scale screens for the identification of anti-CSC compounds.

## METHODS

### Cell lines, monolayer and mammosphere culture

HMLE and HMLER-shEcad cell lines were kindly provided by Dr. Robert Weinberg (MIT, Cambridge, MA, USA) on October 18, 2011, and were cultured as described [13, 51]. The cells were not authenticated afterwards. The MDA-MB-231 cell line was kindly provided by Dr. Nancy E. Hynes, FMI, Basel, Switzerland on July 24, 2011, and tested negative for mycoplasma with MycoProbe (R&D Systems). Cell line authentication was confirmed by short tandem repeat analysis on November 29, 2013, at Microsynth AG to match the fingerprint of the ATCC corresponding cell line. The 4T1-luc2 cell line was purchased from Calliper Life Science (Waltham, MA) on November 18, 2008, and no further authentication was done. Both cell lines were cultured in Dulbecco's Modified Eagle Medium (DMEM, Gibco) with 10% fetal bovine serum (FBS, Invitrogen) and 1% (v/v) antibiotic-antimycotic solution.

Mammospheres were generated by incubating single cell suspensions in serum-free stem cell medium (SCM) containing Mammary Epithelial Cell Growth Medium (MEGM, Lonza, for HMLE and HMLER-shEcad spheres) or Dulbecco's Modified Eagle Medium: Nutrient Mixture F-12 (DMEM/F12, Gibco, for MDA-MB-231 and 4T1-luc2 spheres) supplemented with 2% (v/v) B27 (Gibco), 20 ng/ml epidermal growth factor (EGF, Peprotech Inc.), 20 ng/ml basic fibroblast growth factor (bFGF, Peprotech Inc.), 10 μg/ml insulin, 20 μg/ml hydrocortisone (Sigma-Aldrich) and antibiotic-antimycotic solution as described [13, 51] in flasks coated with poly (2-hydroxyethyl methacrylate) (poly-HEMA, Polysciences Inc.) solution. The spheres were passaged every 7-9 days.

### Chemical libraries and reagents

Two commercially available chemical libraries, the Prestwick chemical library (http://www.prestwickchemical.com/) and the NCI-DTP diversity Set II https://dtp.cancer.gov/databases_tools/data_search.htm were used. Compounds were solubilized at 1 mM in dimethyl sulfoxide (DMSO) and all compounds were diluted in assay media for a final concentration of 10 μM in the screen. The concentration of DMSO in each assay well, including all control wells was 1%. NSC42199 was kindly provided by the NCI/NIH, while Prestw-1013 and Prestw-1236 were purchased from Prestwick Chemical Inc. All other compounds used in *in vitro* assays were bought from Sigma and dissolved in DMSO.

### Chemical screening and data analysis

The chemical screening was performed in a 96-well plate format. 1,000 cells isolated from HMLE and HMLER-shEcad cells were inoculated in a mixed medium of MEGM and DMEM at adherent conditions, while 3,000 sphere-forming cells isolated from related spheres were grown in SCM as suspension. After 24 h, cells were treated with compounds from the chemical libraries at 10 μM or DMSO only. Cell viability was determined by the cell counting kit-8 (CCK-8) assay after 72 h treatment according to the manufacturer's instructions. The optical density (OD) at 450 nm was measured by a microplate reader (Tecan Inc). The cell viability fraction (%) was calculated as follows: OD450nm_-test compound_ / OD450nm_-DMSO_ × 100%. The screening was done for two independent replicates and the quality of the experiments was determined by principal component analysis and calculation of the Pearson correlation coefficients (*r*) of biological replicates using Prism 5.0.

### Chemotherapy sensitivity assays

1,000 cells were seeded in 96-well plates, and various concentrations of paclitaxel or doxorubicin were added after 24 h, and co-incubated for 72 h. The cell viability was measured using the CCK-8 assay.

### Flow cytometry (FACS) analysis for CD44 and CD24

All antibodies were obtained from BD Biosciences (San Diego, CA, USA). Combinations of fluorochrome-conjugated monoclonal antibodies against human CD44 (APC; cat. # 559942) and CD24 (PE; cat. # 555428) or their respective isotype controls (APC mouse IgG2b,κ: cat. #555745; PE mouse IgG2a,κ: cat. #555574) were added to single cell suspensions of MDA-MB-231 spheres at 1:20 and incubated at 4°C for 30 min. The labeled cells were washed in FACS buffer (2 mM EDTA, 1% (w/v) BSA in PBS) twice, and then acquired with a FACS Canto (BD Biosciences). Data were analyzed with Flow Jo software (FlowJo X 10.0.7) and illustrated as percentage of cells with a CD44^+^/CD24^−^ phenotype±SD.

### Aldefluor assay

The ALDEFLUOR assay (STEMCELL Technologies) was used to profile stem and progenitor cells based on their high expression of ALDH1. The Aldefluor assay was conducted according to the manufacturer's instructions. Briefly, single cell suspensions from treated or untreated tumor cells were harvested, washed with Aldefluor assay buffer, the cell density was adjusted to 10^6^ cells/ml in Aldefluor assay buffer supplement with ALDH substrate and cells were incubated for 40 min at 37°C to allow substrate conversion. As a negative control for all experiments, an aliquot of Aldefluor-stained cells was immediately quenched with diethylaminobenzaldehyde (DEAB), a specific ALDH inhibitor. Cells were analyzed using the FITC channel on the FACS Canto. Data were analyzed with FlowJo software. The ALDH^+^ fraction was calculated based on the disappearance of that fraction in the presence of DEAB using the formula: ALDH^+^ fraction = ALDH^+^ percentage_(-DEAB)_ - ALDH^+^ percentage_(+DEAB)_

### Mammosphere formation assay

Mammosphere formation assays were performed as described, but with addition of 0.5% methylcellulose to prevent cell aggregation [18]. 1,000 cells were seeded per well in ultra-low attachment 96-well plates with SCM. After incubation with test compounds for 6 days, the mammosphere numbers (diameter > 50 μm) were counted and photographed.

### Self-renewal assay

Adherent cells were pretreated with compounds or 0.1% DMSO for 4 days. 1,000 cells were dissociated and seeded in ultra-low attachment 96-well plates with 100 μl of SCM. Cells were seeded in parallel at the same density in 6-well plates. 6 days later, the primary mammospheres formed in 96-well plates were counted and photographed. The cells in 6-well plates were dissociated into single cells and seeded as next generation of mammospheres in both 96-well and 6-well plates without treatment. The mammosphere number was measured in different generations of spheres without treatment.

### RNA extraction and quantitative real-time PCR (qRT-PCR)

Total RNA was isolated from cells or sphere-containing pellets using the Nucleo Spin RNA kit (Macherey-Nagel AG) and cDNA synthesis was performed by the High Capacity cDNA Reverse Transcription Kit (Applied Biosystems). qRT-PCR was run in an Applied Biosystems 7900HT fast real-time PCR machine. qRT-PCR reactions were carried out with SYBR Green PCR Master Mix (Applied Biosystems) and ATCB (β-actin) levels were used as controls. The mean cycle threshold value (Ct), normalized to the Ct value of the housekeeping gene (*ATCB*) was used to calculate gene expression values. Primers for human genes were custom-made oligonucleotide primers (Microsynth, Switzerland). Primer sequences are shown in Table S1. Data are given as 2^−ΔΔCt^.

### *In vivo* liming dilution assay

Animal studies were carried out according to the ethical guidelines established by our Institution (ETH Zürich), under approved animal protocols (11/2012 and 12/2015) by the Veterinäramt des Kantons Zürich. Mice were housed in microisolator cages and in pathogen free conditions. Surgical procedures were performed under anesthesia and all efforts were made to minimize suffering of the animals. 4T1-luc2 spheres were maintained in ultra-low attachment plates with SCM and pretreated for 6 days with benztropine mesylate (5 μM) or DMSO (0.1%) *in vitro*. Single cells were injected in 50 μl 1:1 matrigel:DMEM/F12 solution into the fourth mammary fat pad of 8-week old female Balb/c mice (Janvier) at varying cell numbers ranging from 10 to 1,000 cells/mouse. The tumor-initiating frequency, determined four weeks after injection, was used for calculation of frequency of BCSCs using the extreme limiting dilution assay (ELDA) webtool (http://bioinf.wehi.edu.au/software/elda) as described previously [52].

### Statistics

Data are represented as mean±SD. Statistical tests were performed with GraphPad Prism V5.0 (San Diego, CA). A two-tailed Student's t-test was used for comparisons of continuous variables between two groups. One-way ANOVA with Tukey or Dunnett post tests or two-way ANOVA was used when three or more groups were compared.

## SUPPLEMENTARY METHODS, SUPPLEMENTARY FIGURES AND TABLES



## ABBREVIATIONS

ALDH: aldehyde dehydrogenase
BCSCs: breast cancer stem cells
bFGF: basic fibroblast growth factor
CCK-8: cell counting kit-8
CSCs: cancer stem cells
Ct: cycle threshold
DEAB: diethylaminobenzaldehyde
DMEM: Dulbecco's Modified Eagle Medium
DMEM/F12: Dulbecco's Modified Eagle Medium: Nutrient Mixture F-12
DMSO: dimethyl sulfoxide
EGF: epidermal growth factor
ELDA: extreme limiting dilution assay
EMT: epithelial-mesenchymal transition
FBS: fetal bovine serum
mAChRs: muscarinic acetycholine receptors
MEGM: Mammary Epithelial Cell Growth Medium
nAChRs: nicotinic acetylcholine receptors
OD: optical density
poly-HEMA: poly (2-hydroxyethyl methacrylate)
qRT-PCR: quantitative real-time PCR
*r*: Pearson correlation coefficients
SCM: serum-free stem cell medium
