# Supplementary material for: New use of an old drug: inhibition of breast cancer stem cells by benztropine mesylate
Source: Oncotarget. 2016 Nov 24;8(1):1007–22. doi: 10.18632/oncotarget.13537 (PMC5352030; doi:10.18632/oncotarget.13537)
Supplement: Supplementary file 1 [file oncotarget-08-1007-s001.pdf]

New use of an old drug: inhibition of breast cancer stem cells by benztropine mesylate

SUPPLEMENTARY METHODS

Ligand-based target prediction using SPiDER 1.0

For ligand-based target prediction of benztropine mesylate, we used the self-organizing map-based prediction of drug equivalence relationships (SPiDER version 1.0; modlab-cadd.ethz.ch/software/spider/) ((1) Briefly, benztropine mesylate was preprocessed with the Molecular Operating Environment (MOE; Chemical Computing Group, Montreal, Canada; Version 2011.10) "wash" function (protonate bases, deprotonate acids, add hydrogens). Subsequently, the molecule was described in terms of its pharmacophoric correlation vector (CATS2) (2) and physicochemical properties (MOE2D). Using these descriptor vectors, the molecule was co-clustered with known drug-like compounds (COBRA database, v12.6, inSili.com) (3) and targets inferred statistically according to the distance to known ligands in chemical space, rationalized by a background distribution of inter-target drug-drug similarities. The two acquired prediction scores (CATS2 and MOE2D) were averaged to yield a consensus score. This consensus score was again evaluated statistically by converting it into a conservative p value according to a background database of prediction scores achieved by random drug-like molecules. These reported p values indicate the confidence in a prediction by putting them into perspective according to target scores that are acquired trough random chemical similarity. Full technical details can be found in the original publication (1).

REFERENCES

1. Reker D, Rodrigues T, Schneider P, Schneider G. Identifying the macromolecular targets of de novo-designed chemical entities through self-organizing map consensus. Proc Natl Acad Sci USA. 2014;111:4067-72.
2. Reutlinger M, Koch CP, Reker D, Todoroff N, Schneider P, Rodrigues T, et al. Chemically Advanced Template Search (CATS) for Scaffold-Hopping and Prospective Target Prediction for 'Orphan' Molecules. Mol Inform. 2013;32:133-8.
3. Schneider P, Schneider G. Collection of Bioactive Reference Compounds for Focused Library Design. QSAR Comb. Sci. 2003; 22:713-8.

SUPPLEMENTARY FIGURES AND TABLES

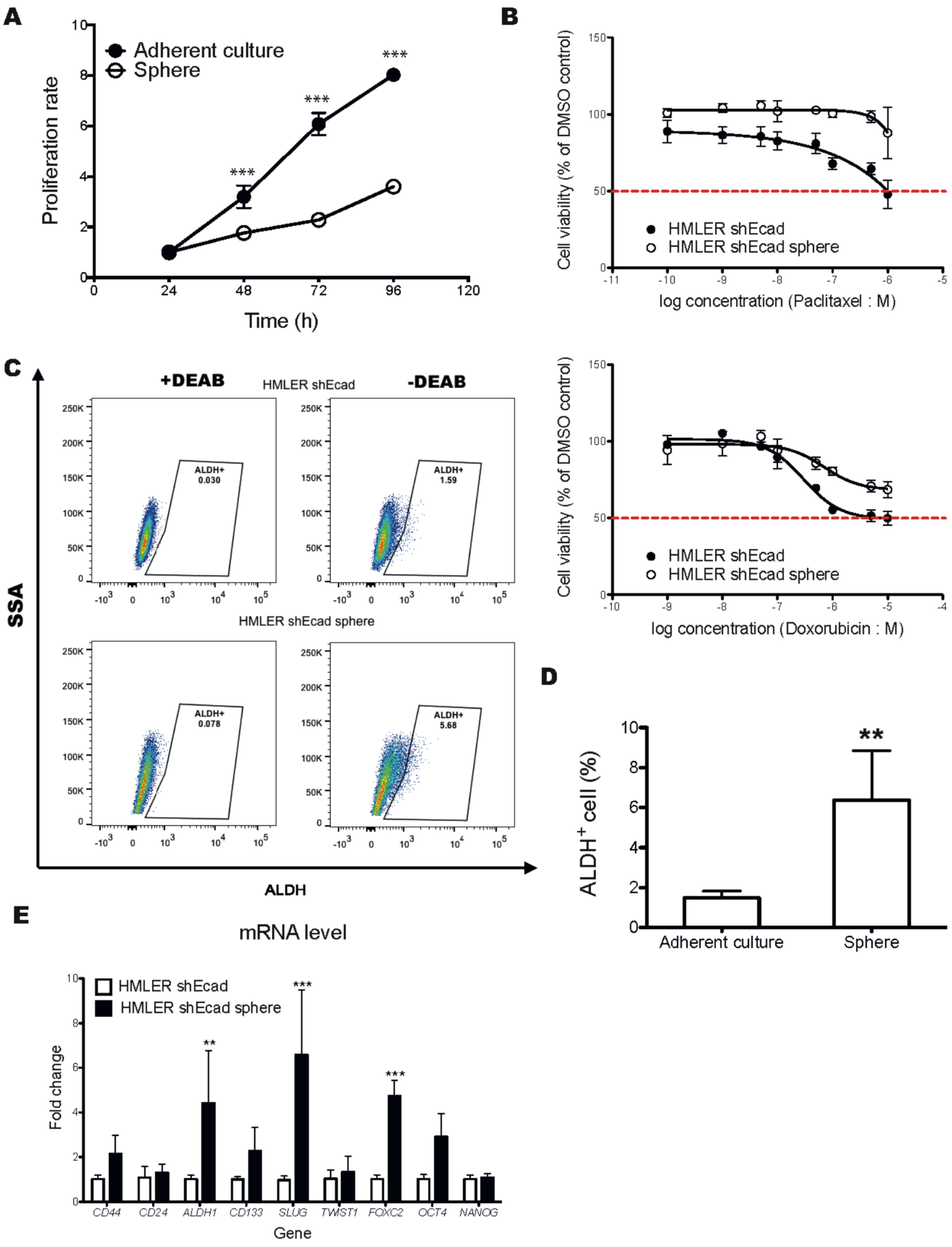

Supplementary Figure S1: Enrichment of BCSCs by mammosphere culture of EMT-induced breast cancer cells (HMLER-shEcad). A. Proliferation curves of 1,000 cells isolated from HMLER-shEcad adherent cells and spheres grown for 96 h. Cell viability was measured by the CCk-8 assay. Data are expressed as mean±SD (n=6). ***p<0.001 (two-way ANOVA). **B.** Dose-response curves of HMLER-shEcad adherent cells and spheres treated with doxorubicin or paclitaxel. **C.** ALDH enzymatic activity in sphere-forming cells compared to adherent cells measured using the ALDEFLUOR kit. **D.** Summary data showing the percentage of ALDH$^+$ cells in the HMLER-shEcad cells and mammospheres. **E.** qRT-PCR analysis of CSC-related genes in HMLER-shEcad adherent cells and spheres. Data are normalized to *ACTB* expression and are presented as fold change in gene expression relative to adherent cells. Data are expressed as mean±SD (n=3). **p<0.01, ***p<0.001 (Student's t-test).

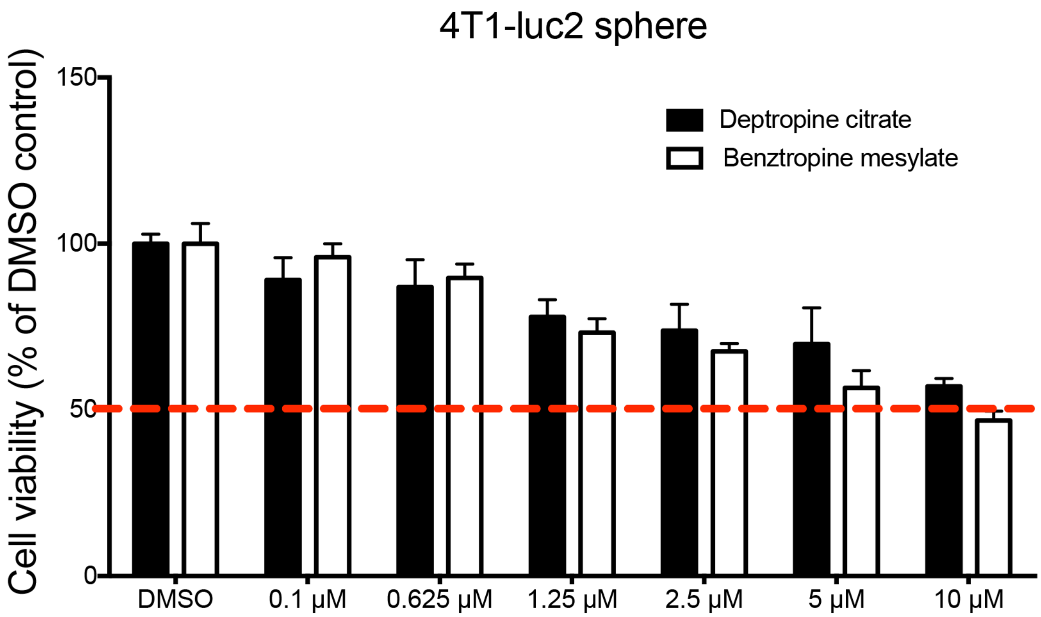

Supplementary Figure S2: Cell viability assay. Cell viability of 4T1-luc2 spheres treated with different concentrations of deptropine citrate and benztropine mesylate for 72 h are shown. Data are expressed as mean±SD.

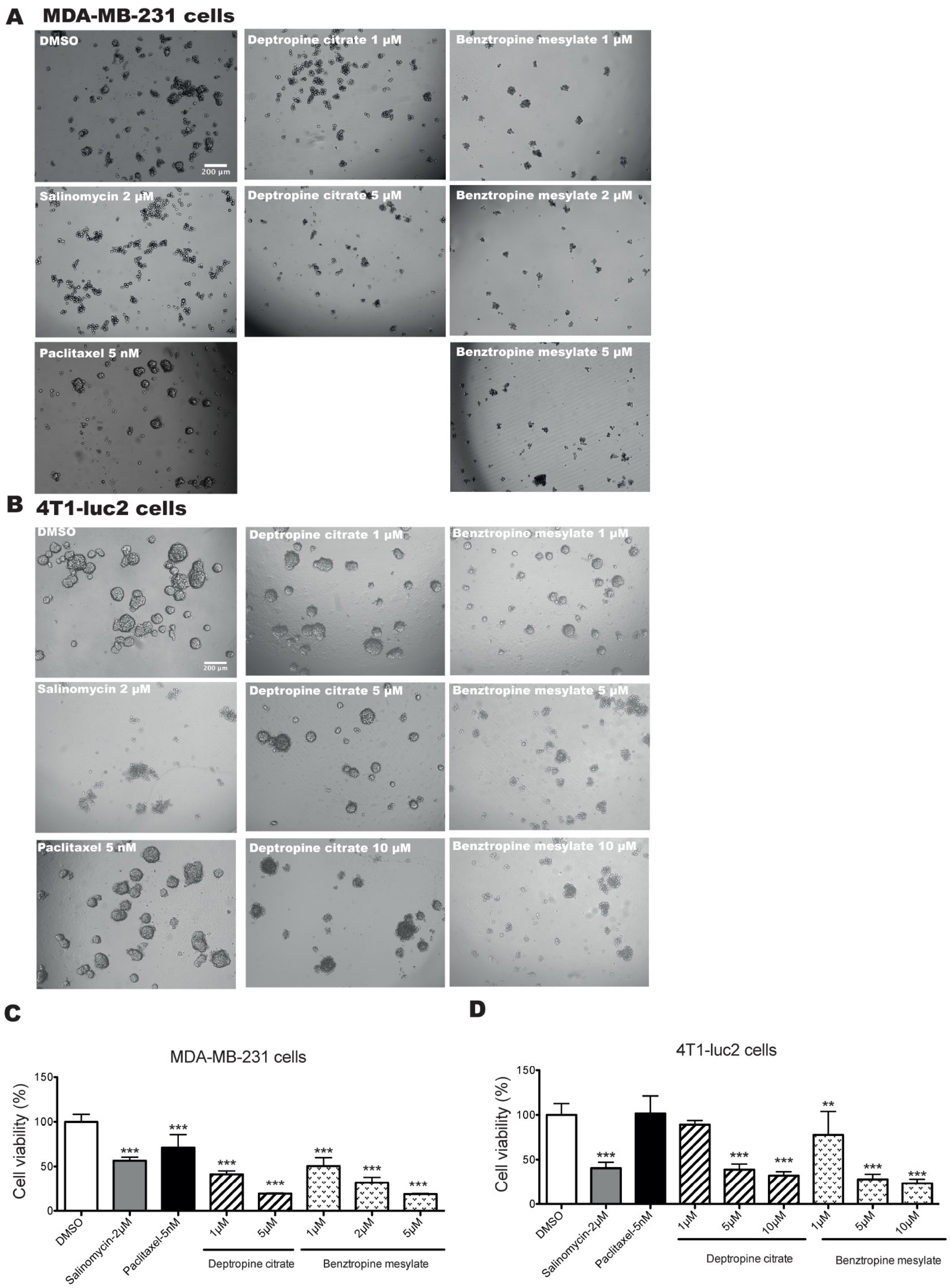

Supplementary Figure S3: Mammosphere formation assay. Morphology **A-B.** and cell viability **C-D.** of mammospheres from MDA-MB-231 cells and 4T1-luc2 cells which were treated with different concentrations of deptropine citrate, benztropine mesylate, salinomycin and paclitaxel for 6 days. Scale bars indicate 200 µm. Data are expressed as mean±SD (*n*=6). $^{**}p<0.01$, $^{***}p<0.001$ compared with DMSO control (one-way ANOVA).

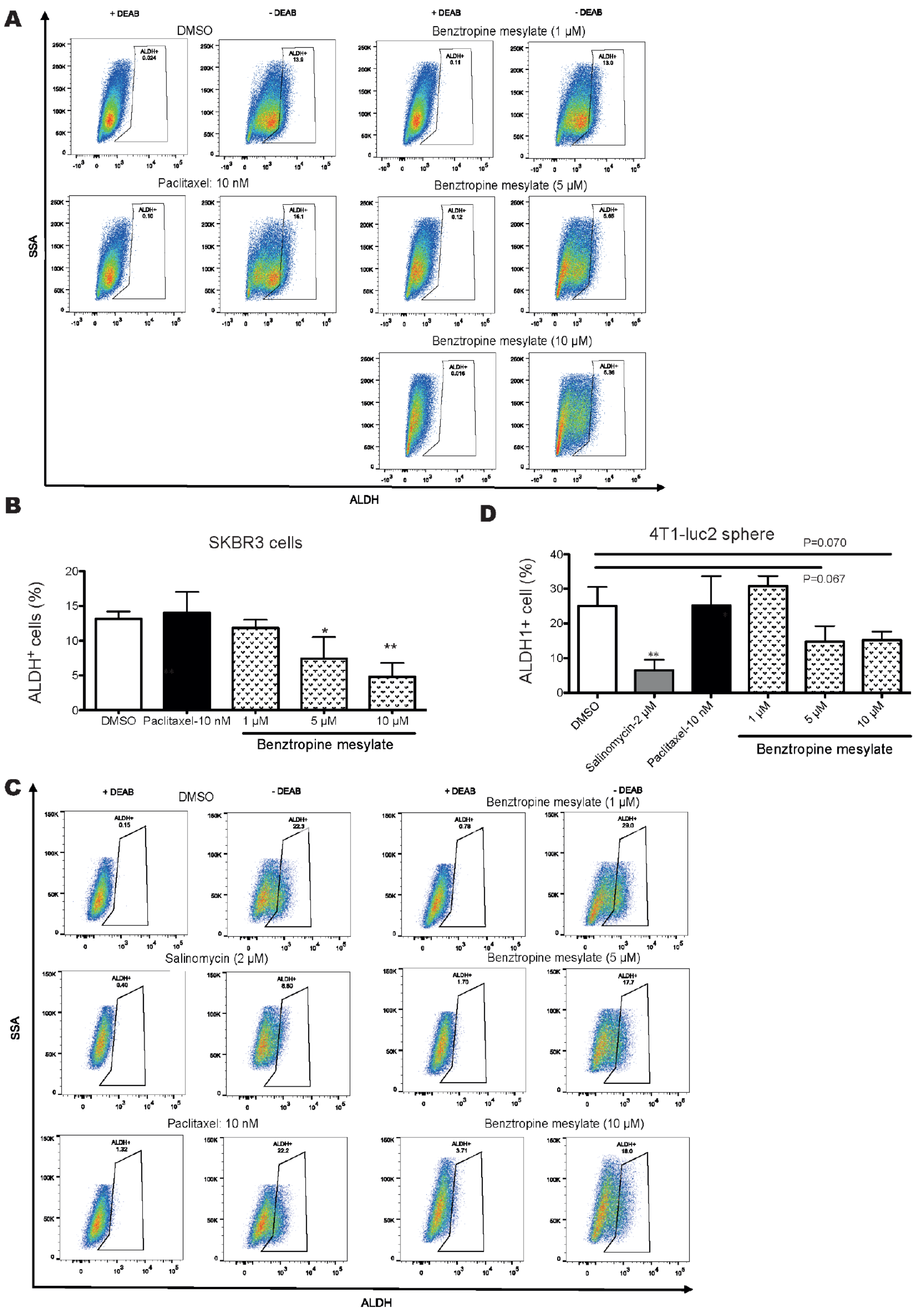

Supplementary Figure S4: FACS analysis of ALDH$^+$ cell population in SKBR3 and 4T1-luc2 cells with or without benztropine mesylate treatment. SKBR3 and 4T1-luc2 cells were treated with benztropine mesylate (1, 5 or 10 μM), paclitaxel (10 nM) or DMSO for 4 days. Single cell suspensions were used for FACS analysis. Representative data for the ALDH$^+$ population in benztropine mesylate-treated SKBR3 cells **A.** and 4T1-luc2 cells **C.** show a reduction compared with DMSO-treated cells. DEAB was used to inhibit the reaction of ALDH with the ALDEFLUOR reagent, providing a negative control. Data of the proportion of ALDH$^+$ cells are shown as mean±SD **B, D.** Experiments (n=3) were conducted in triplicates. *$p < 0.05$, **$p < 0.01$ compared with DMSO control (one-way ANOVA).

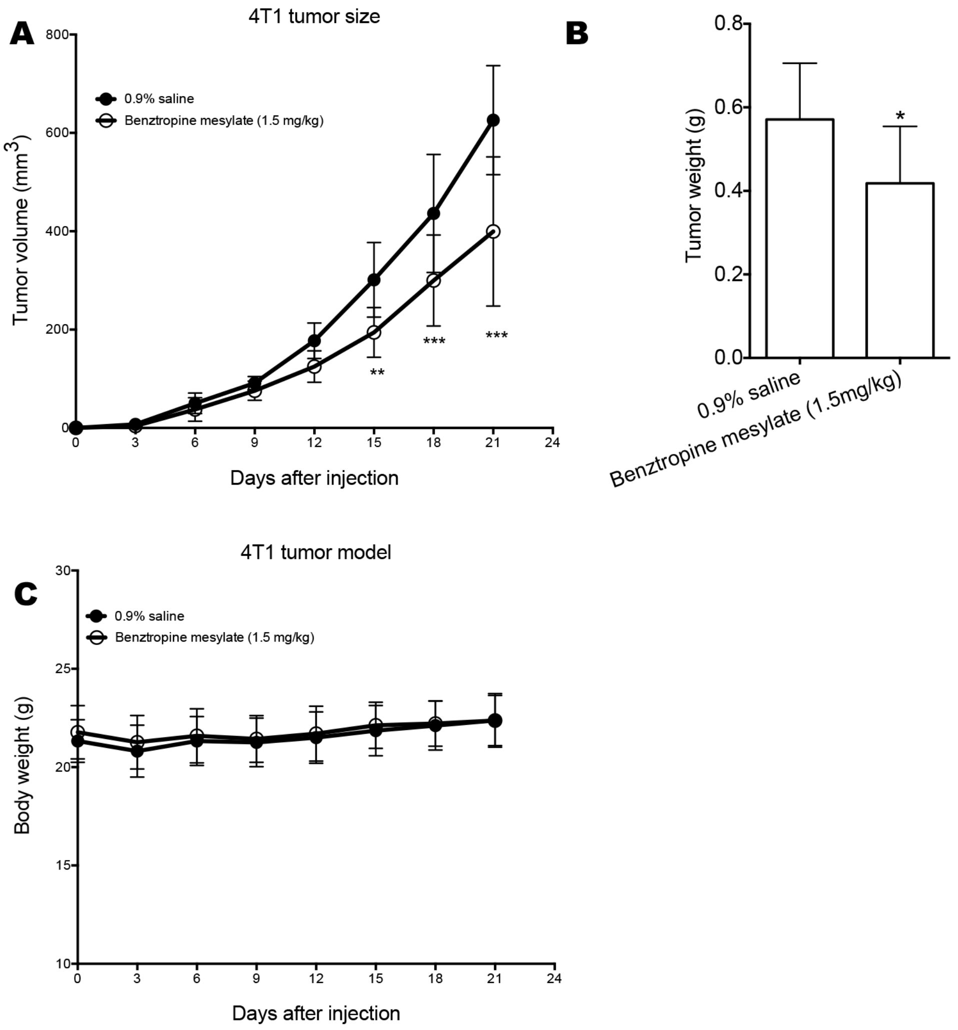

Supplementary Figure S5: Effects of benztropine mesylate treatment on tumor growth *in vivo*. Balb/c mice were injected with 4T1 breast tumors and were treated with benztropine mesylate (1.5 mg/kg; n=10) or 0.9% saline (n=10) for 3 weeks. Tumor volume **A.**, tumor weight **B.**, and body weight **C.** change after treatment. Data are expressed as mean ± SD (n = 10). ***p<0.001, **p<0.01, *p<0.05.

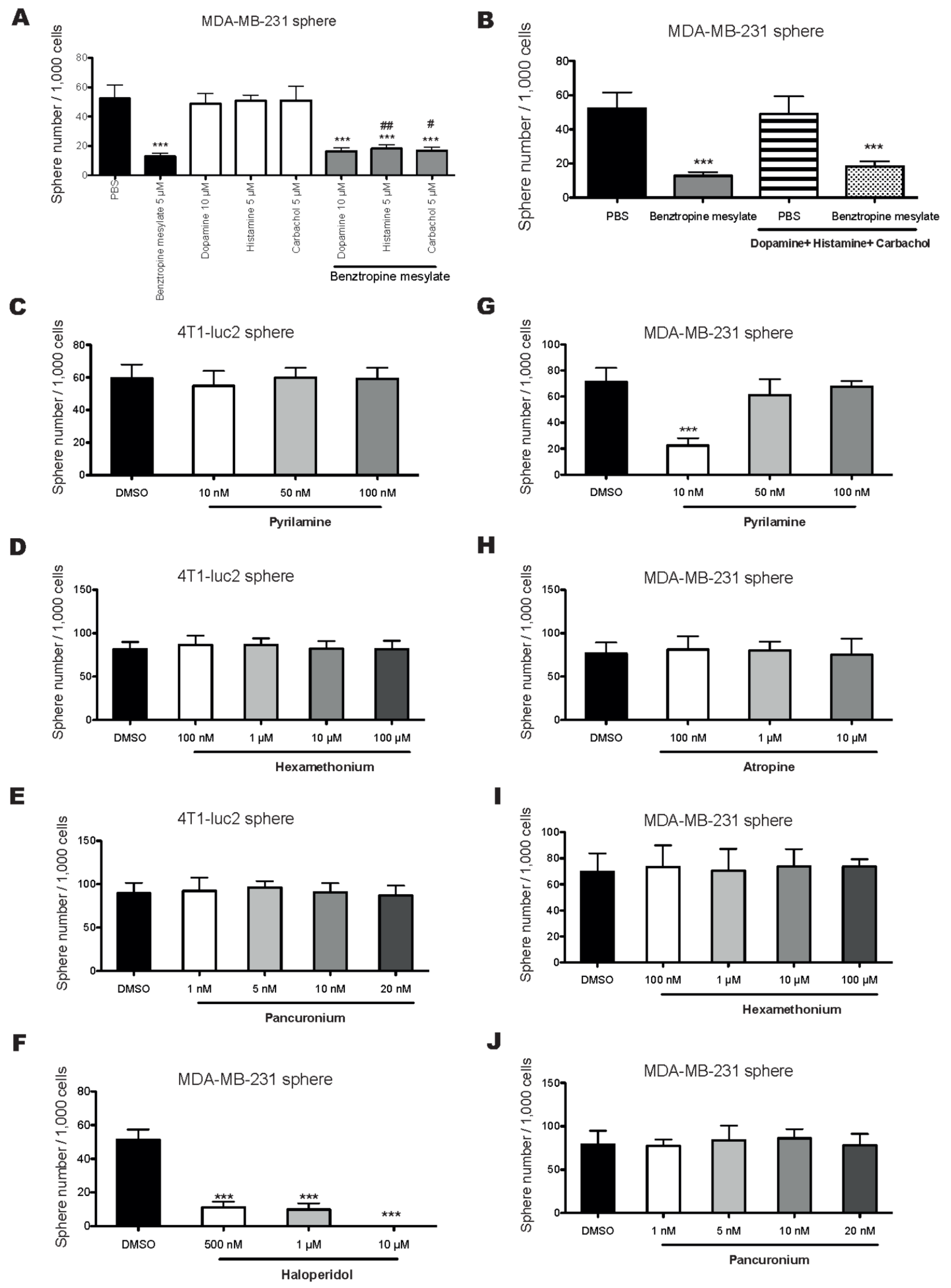

Supplementary Figure S6: Benztropine mesylate partially impairs mammosphere formation of BCSCs through acetylcholine receptors, dopamine receptors/transporters and/or histamine receptors. Qualification of mammosphere formation efficiency of 1,000 MDA-MB-231 cells co-treated with benztropine mesylate (5 μM) and dopamine (10 μM), histamine (5 μM) or carbachol (5 μM) alone or with an agonist combination (dopamine + histamine + carbachol) for 6 days **A-B.** Quantification of mammosphere numbers from 1,000 MDA-MB-231 and 4T1-luc2 cells treated with the histamine receptor antagonist pyrilamine **C, G.**, the nicotine receptor antagonist hexamethonium bromide **D, I.**, the muscarinic receptor antagonist atropine **H.**, the antagonist of both types of receptors pancuronium bromide **E, J.**, or the dopamine receptor antagonist haloperidol **F.** for 6 days. Data are expressed as mean±SD (n=6). ***p<0.01, ***p<0.001 compared with DMSO control (One-way ANOVA).

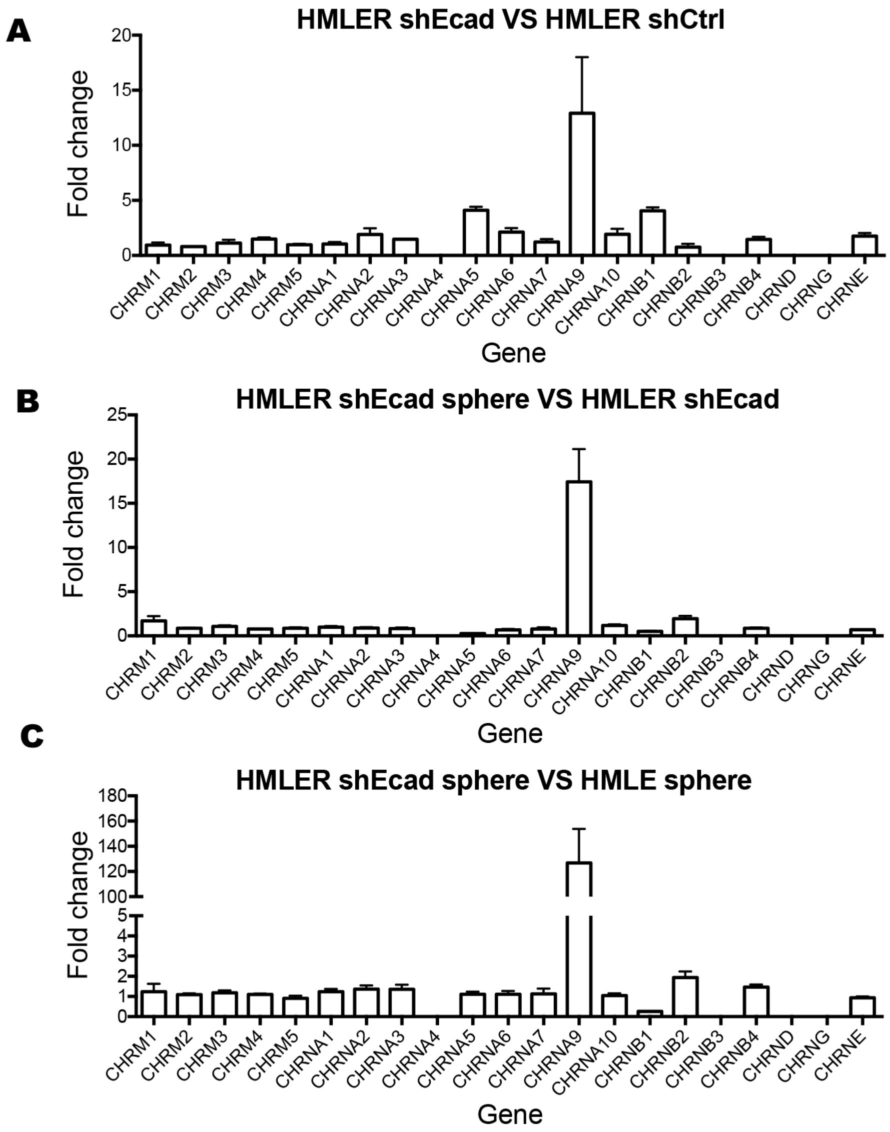

Supplementary Figure S7: Comparison of acetylcholine receptor mRNA expression levels in HMLE, HMLER shCtrl, and HMLER shEcad adherent cells and spheres. Data were normalized to ACTB expression. The fold changes in relative gene expression were compared in **A.** HMLER shEcad adherent cells vs HMLER shCtrl adherent cells, **B.** HMLER shEcad spheres vs HMLER shEcad adherent cells, and (C) HMLER shEcad spheres vs HMLE spheres. Data are expressed as mean ± SD (n = 3).

Supplementary Table S1: Primer sequences for qRT-PCR analysis

Gene	Forward (5'-> 3')	Reverse (5'-> 3')
ACTB	CATGTACGTTGCTATCCAGGC	CTCCTTAATGTCACGCACGAT
CD44	ACCCCAGCAACCCTACTGCTGAT	TAGCAGGGATTCTGTCTGTG
CD24	CTCCTACCCACGCAGATTTATTC	AGAGTGAGACCACGAAGAGAC
ALDH1	CCGTGGCGTACTATGGATGC	GCAGCAGACGATCTCTTTCGAT
CD133	AGTCGGAAACTGGCAGATAGC	GGTAGTGTTGTACTGGGCCAAT
SLUG	TGTGACAAGGAATATGTGAGCC	TGAGCCCTCAGATTTGACCTG
TWIST1	GTCCGCAGTCTTACGAGGAG	GCTTGAGGGTCTGAATCTTGCT
FOXC2	CCTCCTGGTATCTCAACCACA	GAGGGTCGAGTTCTCAATCCC
OCT4	CTTGAATCCCGAATGGAAAGGG	GTGTATATCCCAGGGTGATCCTC
NANOG	TGATTTGTGGGCCTGAAGAAAA	GAGGCATCTCAGCAGAAGACA
CHRM1	CTCTATACCACGTACCTGCTCA	CCGAGTCACGGAGAAGTAGC
CHRM2	AACTCCTCTAACAATAGCCTGGC	GTTCCCGATAATGGTCACCAAA
CHRM3	CACAATAACAGTACAACCTCGCC	GCCAGGATGCCCGTTAAGAAA
CHRM4	GTTTGTGGTGGGTAAGCGGA	TGCTTCATTAGTGGGCTCTTG
CHRM5	CAATGCAACCACCGTCAATGG	ATCTGCACAGGCTAAGCTGAG
CHRNA1	TCCTGGGCTCCGAACATGA	ACATTGGTTGTCACGATCTGATT
CHRNA2	AGGCTCGCATACCGAGACT	TCACCACGTCTGAAGTGTTGG
CHRNA3	TGAGCACCGTCTATTTGAGCG	TGGACACCTCGAAATGGATGAT
CHRNA4	GGAGGGCGTCCAGTACATTG	GAAGATGCGGTCGATGACCA
CHRNA5	AAAGATGGGTTCGTCCTGTGG	CAAACAAAACGATGTCTGGTGTC
CHRNA6	TGAGACTCTTCGCGTTCCTG	ATTTCAGCTTTGTCATACGTCCA
CHRNA7	GCTGGTCAAGAACTACAATCCC	CTCATCCACGTCCATGATCTG
CHRNA9	AAATCTGGCACGATGCCTATC	GCAGGACCACATTGGTGTTCA
CHRNA10	TCGACATGGATGAACGGAACC	ATCGTAGGTAGGCATCTGTCC
CHRNB1	CTCTGGACATTAGCGTCGTGG	GCTGAACACCATAGTGCAATTCT
CHRNB2	GGTGACAGTACAGCTTATGGTG	AGGCGATAATCTTCCCACTCC
CHRNB3	TGCTGGTTCTCATCGTCCTTG	GCATCTTCATTTTCGGCGATTGA
CHRNB4	AACCCGTTACAATAACCTGATCC	ATTCACGCTGATAAGCTGGGC
CHRND	ATGGTCAACCTGGTCTTCTACC	CAGGAACTTGCCGATAAGGG
CHRNG	ACGAGACTCGGATGTGGTCAA	GACACCGTCCACGTTGTTCT
CHRNE	GTGGATGCCGTGAACTTCGT	GCACCCAGTCGGACACTTC

Supplementary Table S2: Target prediction of benztropine mesylate by SPiDER 1.0 software

Rank	Target	*p* value
1	Nicotinic Acetylcholine Receptor Agonist	0.005
2	Voltage Dependent Calcium Channel Antagonist	0.009
3	Voltage-Gated Potassium Channel	0.009
4	Neuropeptide Y 1 (NPY) Antagonist	0.011
5	Sigma Receptor S1 & S2	0.012
6	Histamine Receptor H1 & H2	0.016
7	Opioid Receptor Delta, Mu & Kappa	0.016
8	Aspartic Endopeptidase (Secretase & Renin)	0.018
9	Monoamine Oxidase (MAO)	0.018
10	Dopamine Transporter, 5-HTT, NAT & Glycine Transporter	0.019
11	Proteinase-Activated Receptor (PAR-1)	0.022
12	Leukotriene-A(4) Hydrolase	0.022
13	Muscarinic Acetylcholine Receptor Antagonist (M1, M2, M3)	0.023
14	Dopamine Receptor Antagonist (D1, D2, D3, D4)	0.030
15	Phosphodiesterase 3 & 5	0.030
16	Cannabinoid Receptor 1 & 2	0.032
17	Serotonin Receptor 1-7	0.033
18	Serine Endopeptidase (Factor Xa, Beta-Tryptase, Thrombin)	0.038
19	Sodium Channel	0.039
20	Serine Threonine Kinase (MAPK14, CK1, AKT, ROCK, A, C, Casein I, p38 MAP)	0.040
21	Ghrelin Receptor	0.041
22	GABA Receptor A & C	0.042
23	GABA Transporter I Antagonist	0.043
24	Melanin Concentrating Hormone-1 Receptor Antagonist	0.046
25	Neurokinin 1 & 2 Receptor Antagonist	0.047
